# 

**Published:** 1895-12

**Authors:** 


### The Library of the Bristol Medico-Chirurgical Society.

At the Annual Meeting held on October 9th, 1895, Mr. L. M. Griffiths, the Honorary Librarian, read the following Report:—

Since the last Annual Meeting 754 volumes have been added to the Society's Library, which now consists, including 542 duplicates, of 4,530 volumes. The number of current periodicals regularly received in the Library is 127.

The donors of books during the twelve months have been: Sir Thomas Dyke Acland, Bart., the British Medical Association, Dr. R. E. Burges, Mr. E. F. H. Burroughs, Dr. J. Michell Clarke, Mr. T. F. Edgeworth, Dr. C. Elliott, Mr. L. M. Griffiths, Dr. A. J. Harrison, the Council of the Obstetrical Society of London, Dr. Arthur B. Prowse, Dr. H. D. Rolleston, the Royal Academy of Medicine in Ireland, the Sanitary Publishing Company, Mr. J. Greig Smith, Dr. R. Shingleton Smith, the Surgeon-General United States Army, the Council of University College, Bristol, the Editors of the Pathological Report of University College, London, the Staff of Westminster Hospital, and Messrs. J. Wright & Co. Three framed pictures have been presented by Mr. T. F. Edgeworth, and two unframed by Mr. C. K. Rudge.

Thanks to the combined arrangement under which we are working, the total number of volumes now at the service of members of this Society is 14,328, and in the Library there are altogether 151 periodicals.

Considerable progress has recently been made with the work of cataloguing the Library; and I feel sure that if members will familiarise themselves with the card catalogue they will soon appreciate its advantages.

Largely owing to the co-operation of the authorities of University College, the Library facilities which members of the Society possess are almost unrivalled, and should only require to be fully known to secure a large increase in the number of our members. The advantages of the Library should make it worth the while of all practitioners in the neighbouring towns and villages to join the Society.

The duplicates, forming in themselves a not unimportant library both as regards size and usefulness, are at the service of members, who may take them out on loan.

The attention of members is specially directed to the list of "Desiderata" sent out with the June number of the *Journal*, and copies of which can still be obtained on application to the Clerk. The Library Committee feel that many members of the Society have on their shelves volumes which they rarely or never use, and which would be exceedingly welcome in the Library for the purpose of making up sets.

The Library has now become of so important a character that the Committee think it ought to have a distinctive book-plate or rather book-plates; for there should be one for each of its sections. The dominant idea of the College book-plate should be one symbolising student life, and should emphasise the work of teaching carried on in the Medical School and the Hospitals; while the Society book-plate should bring into prominence some of the kinds of work in which the practitioner is engaged. There are professional artists who are prepared to submit designs for book-plates; but with so much artistic talent amongst the members of the Society we ought to be independent of them and be able to secure appropriate devices from amongst ourselves. I shall gladly receive sketches that could be afterwards reproduced for the object in view. The book-plates should measure 4½ inches laterally and 3½ inches vertically. The provision of sketches for this purpose will undoubtedly give the comic man an opportunity, and in this he should be encouraged, as it would be interesting to form a collection of all those sent in, as no doubt most of them would display considerable artistic ingenuity. Library matters, like all matters medical, are getting specialised. Not only is there a journal—*The Library*—which considers all questions of library management and detail, but there is a monthly magazine—*The Journal of the "Ex Libris" Society*—which deals exclusively with the subject of book-plates, and which may possibly furnish hints to any who stand in need of them.

---

*The following donations have been received since the publication of the list in September:*

*November 30th, 1895.*

| | |
|---|---|
| British Medical Association (1) | 28 volumes. |
| C. Elliott, M.D. (2) | 1 volume. |
| L. M. Griffiths (3) | 1 " |
| Stephen Paget (4) | 1 " |
| Edward Picton Phillips (5) | 6 volumes. |
| Sanitary Publishing Company (6) | 1 volume. |
| J. Greig Smith (7) | 1 " |
| R. Shingleton Smith, M.D. (8) | 11 volumes. |
| Council of University College, Bristol (9) | 1 volume. |
| Editors of the Pathological Report of University College, London (10) | 1 " |

Unbound periodicals have been received from the British Medical Association and Dr. R. Shingleton Smith.

### EIGHTEENTH LIST OF BOOKS.

The titles of books mentioned in previous lists are not repeated.

The figures in brackets refer to the figures after the names of the donors, and show by whom the volumes were presented. The books to which no such figures are attached have either been bought from the Library Fund or received through the *Journal.*

---

**Barling, G.** ... ... *On Appendicitis, and on Perforation of Gastric and Duodenal Ulcer* ... ... ... ... ... ... ... ... 1895

**Bell, J.** ... ... *Notes on Surgery for Nurses* ... ... ... ... ... 4th Ed. 1895

**Bourneville et J. Noir** *Recherches sur l'Épilepsie et l'Idiotie* ... ... ... ... ... 1895

**Brodhurst, B. E.**... *Congenital Dislocation of the Hip* ... ... ... ... ... ... 1895

,, ,, ,, ,, ... ... ... 2nd Ed. 1895

**Carter, A. H.** ... *Elements of Medicine* ... ... ... ... ... ... 7th Ed. 1895

**Cheyne, W. W.** ... *Tuberculous Disease of Bones and Joints* ... ... ... 1895

**Crichton-Browne, Sir J.** *On Dreamy Mental States* ... ... ... ... ... 1895

**Curling, T. B.** ... *Diseases of the Rectum* ... ... ... ... (5) 2nd Ed. 1855

**Grandin and G. W. Jarman, E. H.** *Pregnancy, Labor, and the Puerperal State* ... ... ... ... ... ... ... ... ... ... ... 1895

**Hart, Mrs. E.** ... *Diet in Sickness and in Health* ... .. ... ... ... 1895

**Hutchinson, J.** ... *Smaller Atlas of Clinical Illustrations* ... ... ... ... 1895

**Jarman, E. H. Grandin and G. W.** *Pregnancy, Labor, and the Puerperal State* .. ... ... ... ... ... ... ... ... ... ... 1895

**Lindsay, R.** ... ... *Malaria* ... ... ... ... ... ... ... ... ... ... ... ... 1895

**Macnamara, N. C.** *Regulations as to Defects which disqualify for Admission into Government Services* ... ... ... ... ... ... 1895

**Marsh, H.** ... ... *Diseases of the Joints and Spine* ... ... ... ... ... ... 1895

**Martin, S.** ... ... *Diseases of the Stomach* ... ... ... ... ... ... ... ... 1895

**Moor, T. H. Pearmain and C. G.** *Aids to the Analysis of Food and Drugs* [1895]

**Morris, H.** ... ... *Injuries and Diseases of the Genital and Urinary Organs* 1895

**Newman, G.**... ... *On the History of the Decline and Final Extinction of Leprosy as an Endemic Disease in the British Islands* ... ... ... ... ... ... ... ... ... ... 1895

**Noir, Bourneville et J.** *Recherches sur l'Épilepsie et l'Idiotie* ... ... ... 1895

**Paget, [Sir] J.** ... *Records of Harvey* ... ... [Reprint of 1846 Ed.] (4) 1887

**Pearmain and C. G. Moor, T. H.** *Aids to the Analysis of Food and Drugs* [1895]

**Richardson, B. W.** *Clinical Essays. Asclepiad,* Vol. I. ... ... ... ... ... 1862

**Snell, S.** ... ... *Eyesight and School Life* ... ... ... ... ... ... ... 1895

**Stephenson, S.** ... *Epidemic Ophthalmia* ... ... ... ... ... ... ... ... 1895

**Stewart, G. N.** ... *A Manual of Physiology*... ... ... ... ... ... ... ... 1895

**Stocken, J.** ... ... *Dental Materia Medica.* 4th Ed.(Ed. by L. M. Stocken and J. O. Butcher) ... ... ... ... ... ... ... 1895

*Tallerman-Sheffield Patent Localized Hot-Air Bath, The*... ... ... ... ... 1895

**Turnbull, J.**... ... *On Consumption* ... ... ... ... ... ... (5) 2nd Ed. 1850

**West, C.** ... ... *Diseases of Women* ... ... ... ... ... ... (5) 3rd Ed. 1864

### TRANSACTIONS, REPORTS, JOURNALS, &c.

Academy of Medicine in Ireland, Transactions of the
Vols. I., 1883, IV., V., 1886-87, VII. 1889
American Journal of the Medical Sciences, The
Vols. LXXXI.—LXXXV., 1881-83; LXXXVII. 1884
American Surgical Association, Transactions of the ... ... Vol. VI. 1888
Annals of Surgery ... ... ... ... ... ... ... .. ... (7) Vol. XI. 1890
Archives of Surgery ... ... ... ... ... ... ... ... ... ... Vol. V. 1894
Asclepiad, The ... ... ... ... ... ... ... ... (1) Vols. I.—III. 1884-86
British Gynæcological Journal, The ... ... ... ... ... (3) Vol. IV. 1888
Buffalo Medical and Surgical Journal ... ... ... ... ...Vol. XXXIV. 1895
Calendar of University College, Bristol ... ... ... ... ... ... (9) 1895
Centralblatt für Chirurgie ... ... ... ... ... ... ... ... ... ... 1894
Centralblatt für Gynäkologie ... ... ... ... ... ... ... ... ... ... 1894
Clinical Journal, The ... ... ... ... ... ... ... ... ... Vol. VI. 1895
Clinical Sketches ... ... ... ... ... ... ... ... ... ... Vol. I. 1895
Clinical Society of London, Transactions of the ... Vols. I.—VII. 1868-74
Diphtheria Cultures, Report of the Medical Officers of Health of Bristol on the Bacteriological Examination of ... ... ... ... ... ... ... 1895
Edinburgh Medical Journal (8) Vols. XXIII., Part 2—XXV., 1878-80,
XXIX., Part 2—XXXII., Part 1, 1884-87
Edinburgh Obstetrical Society, Transactions of the
Sessions 1868-71. 2 vols. 1870-72
Grant College Medical Society, Bombay, Transactions of the, 1894 ... 1895
Hospital, The ... ... ... ... ... ... ... ... ... ... Vol. XVIII. 1895
Hospitals—

- Guy's Hospital Reports ... ... ... ... ... ... ... Vol. LI. 1895
- Johns Hopkins Hospital Reports, The ... ... ... ... Vol. III. 1894
- University College, London—Pathological Report (10) Vol. V. [N.D.]

Intercolonial Medical Congress of Australasia, Transactions of the 3rd Session, 1892 ... ... ... ... ... ... ... ... ... ... ... (1) 1893
International Clinics ... ... ... ... ... ... ... (1) 4th Series, Vol. I. 1894
Journal of the American Medical Association, The ... ... Vol. XXIV. 1895
Library Association of the United Kingdom, Report of Proceedings of the 16th and 17th Annual Meetings of the, 1893, 1894. 2 vols. ... ... 1895
Library Association Year Book, The ... ... ... ... ... ... ... ... 1895
Liverpool Medico-Chirurgical Journal, The ... ... Vols. I., II., 1857-58
London Medical Recorder, The ... ... ... ... ... ... (2) Vol. III. 1890
Marine-Hospital Service of the United States, Annual Report of the Supervising Surgeon-General of the, for 1892 ... ... ... ... ... (1) 1893
Medical Chronicle, The ... ... ... ... ... ... ... N.S., Vol. III. 1895
Medical Pioneer, The ... ... ... ... ... ... ... ... ... Vol. I. 1893
Medico-Chirurgical Transactions (1) Vols. LXXIII., 1890; LXXVIII. 1895
Montreal Medical Journal, The ... ... ... ... ... ... Vol. XXIII. 1895
Navy, Statistical Report of the Health of the, for 1892 ... ... ... (1) 1893
New England States, Vital Statistics of the, for 1892 ... ... ... ... [N.D.]
Ophthalmological Transactions ... ... ... ... ... ... Vol. XV. 1895
Quarterly Medical Journal, The ... ... ... ... ... ... ... Vol. III. 1895
Registrar-General of Births, Deaths, and Marriages in England—Reports
(1) LV., LVI. 1894

Registrar-General of Births, Deaths, and Marriages in Scotland, 37th Detailed Annual Report of the (Abstracts of 1891) ... ... ... (1) 1893
Registrar-General of Marriages, Births, and Deaths in Ireland, 29th Detailed Annual Report of the, 1892 ... ... ... ... ... ... (1) 1893
Royal Medical and Chirurgical Society of London, Proceedings of the Vols. I.—IX., 1857-82; N.S., Vols. I., II., 1885-88
St. Louis Medical and Surgical Journal, The ... ... Vol. LXVIII. 1895
Sanitary Institute of Great Britain, Transactions of the (1) Vols. XII.—XIV., 1892-94
Sanitary Record, The ... ... ... (6) Vols. XIV., 1893; XV., XVI., 1894-95
Sei-I-Kwai Medical Journal, The ... ... ... ... ... ... Vol. XIII. 1894
University Medical Magazine ... ... ... ... ... ... Vol. VII. 1895
West London Medico-Chirurgical Society, Proceedings of the (1) Vols. I., 1884, IV., 1891; VI. 1895
Year-Book of Treatment, The ... ... ... ... ... ... ... 1888, 1890

At p. 235, for "Lewis, H. L.," read "Jones, H. L."

### Local Medical Notes.

BRISTOL HEALTH REPORTS.

At the commencement of the year the Public Health Department undertook the bacteriological examination of all cases of supposed diphtheria occurring in the city, with a view of confirming the clinical diagnosis, which must in many cases be more or less tentative. In July Dr. Davies presented a report on the work of the first six months. We were unable to give an earlier notice of this, as our last number was in the printer's hands when the report reached us. We believe that our city was one of the first in this country to undertake this sort of work, and the reports of the examinations fully justify the step taken. During the six months ending June 30th 73 cases were notified to the Medical Officer of Health; two of these were subsequently decided on clinical grounds by the medical attendant not to be cases of diphtheria. Of the 71, 29 were examined, and the Löffler bacillus found in 24; in the remaining 42 the medical men did not send inoculated tubes, though eleven were examined, with five negative results. It is to be hoped that a larger proportion will be sent in the future, as we believe the word diphtheria is used somewhat rashly. Second and third examinations were made in some cases, at varying intervals, with the result that the bacillus was found in two cases to be present twenty-five and twenty-eight days respectively. These examinations are of the greatest value, as the period of quarantine after diphtheria is by no means accurately fixed. Two very interesting cases are reported, in which the bacillus was found in the disease known as fibrinous rhinitis, the identity of the micro-organism being confirmed by Dr. Klein, to whom a specimen was sent, and further by the mother of the patients contracting diphtheria presumably from her children. Dr. Dowson, who made the examinations at the laboratory of the Public Health Department, has endeavoured to discover whether the bacillus can be shown to exist as a saprophyte under certain insanitary conditions. Unfortunately his experiments have not led to any results, as no growth appeared in the tubes inoculated from foul drains or manure heaps. Certain animals—hens, a turkey, and cats—from infected houses were also examined, but with negative results, except in the case of two cats, which undoubtedly suffered from diphtheria. Commenting on these cases, Dr. Dowson expresses his opinion that an enquiry might well be made into the conditions under which domestic animals are kept, especially in the cases of small tradespeople. The report is interesting, and marks a new departure in our Public Health Department.

It is with mingled feelings of surprise and regret that we learn that certain remarks made in our last number have been construed to mean that the Public Health Department of this city is extravagant in its expenditure. After reading the paragraph over again, we are still unable to see how such a construction can be put upon our words. Nothing was further from our thoughts. No one can deny that our rates are high, but to charge the Public Health Department with unnecessarily raising them by extravagant expenditure was not our intention, and in our opinion is not implied by our words. We print below an analysis of the General District Rate, or as it is often, but erroneously, called the Sanitary Rate :—

| | | | |
|---|---|---|---|
| Finance ... ... ... ... ... ... | 3.58 | pence in £ | for half-year. |
| Highways, Sewers and Cleansing | 8.56 | ,, | ,, |
| Parks and General Purposes ... | 2.99 | ,, | ,, |
| Public Health ... ... ... ... ... | .89 | ,, | ,, |
| Street Improvement Committee... | 4.15 | ,, | ,, |
| Baths and Washhouses Committee | .26 | ,, | ,, |
| Electrical Committee ... ... ... | .49 | ,, | ,, |
| Museum Committee ... ... ... | .24 | ,, | ,, |

From this it will be seen that the Department in question is one of the least expensive ones under the Sanitary Authority, requiring less than a penny in the pound. There are matters connected with the health of the city, such as sewers, cleansing, destruction of rubbish, and urinals, which come under the Sanitary Authority; but these are not included in the Public Health Department and are not under the control of the Medical Officer of Health. These, it will be seen, cost eightpence-halfpenny. We print also a statement of the cost of the Public Health Department in seven large towns where the circumstances are much the same as ours, and it will be seen that the expenditure in our city is less than in all but one:—

COMPARATIVE STATEMENT

*Of the Cost of the Medical Officer's Department, Hospital Maintenance, and Port Sanitary Expenses in several large towns for the year ending 25th March, 1895.*

| Town. | Population. | Cost of Med. Officer's Department, Disinfecting and Health Inspection. | Maintenance of Hospitals. | Port Sanitary Working Expenses. | Total. | Equivalent to a rate of in the £. |
|---|---|---|---|---|---|---|
| | | £ s. d. | £ s. d. | £ s. d. | £ s. d. | d. |
| Liverpool ... | 517,980 | 8,441 8 2 | 16,124 18 10 | 1,433 1 6 | 25,999 8 6 | 2.09 |
| Birmingham .. | 478,113 | 6,273 12 0 | 18,618 10 11 | ... ... | 24,892 2 11 | 2.95 |
| Leeds ... ... | 367,505 | 7,366 17 2 | 7,167 18 9 | ... ... | 14,534 15 11 | 2.72 |
| Sheffield ... | 324,291 | 3,363 14 3 | 10 230 16 6 | ... ... | 13,594 10 9 | 3.13 |
| Bristol ... ... | 221,578 | 4,647 19 0 | 3,354 6 10 | 1,424 12 7 | 9,426 18 5 | 2.22 |
| Bradford ... | 216,361 | 6,051 3 6 | 6,160 1 6 | ... ... | 12,211 5 0 | 2.7 |
| Hull ... ... | 200,044 | 3,824 12 3 | 5,326 10 2 | 2,069 10 8 | 11,220 13 1 | 4.14 |

### UNIVERSITY COLLEGE, BRISTOL.

R. Shingleton Smith, M.D. Lond., F.R.C.P. Lond., has been made Emeritus Professor of Medicine.

In the Faculty of Medicine the entries this year amount to 19, with 10 in the Faculty of Arts for the Preliminary Scientific Examination for the University of London.

The Annual Distribution of Prizes to the successful students of the Faculty of Medicine took place in the Theatre of the Museum and Library on November 20th. The Bishop of Hereford, as President of the College, occupied the Chair, and after a few words requested Mr. Nelson C. Dobson to take his place and present the prizes and certificates. With the assistance of the Dean of the Faculty, this ceremony was quickly performed, midst the usual uproarious accompaniment which medical students consider necessary on these occasions. Mr. Dobson then gave an excellent address to the students, reminding them of the seriousness of the profession they had adopted, and especially dilating on the duties that they will have to perform, when qualified, to their patients, to their profession, and to the public. The address was listened to with marked attention and with more respect from the students than is usual; and with a vote of thanks to Mr. Dobson, proposed by Mr. Albert Fry and seconded by Mr. P. J. Worsley, the proceedings terminated.

The following students have recently passed examinations :—

**University of London.** — M.B. Examination, October, 1895. Pass List :—*Second Division*—J. H. Bodman.

**University of Durham.**—Examination for the degree of Bachelor of Medicine. First and Second Examination (with 2nd Class Honours.) —R. F. Moorshead.

**Examining Board in England by the Royal Colleges of Physicians and Surgeons** —Four Years Curriculum. October. *Materia Medica*—H. L. W. Norrington. Five Years Curriculum. Part I. *Chemistry and Physics* —K. H. Beverley. *Elementary Biology*—A. R. M'Ennery. *Elementary Anatomy*—A. M. S. Tordiffe. Part II. *Anatomy and Physiology*—F. A. Coates, E. S. Edwards, G. H. Irvine, J. W. Wallace. *Physiology*—G. L. Wright.

**Royal College of Physicians.**—Final Examination—W. T. Lydall, E. W. Ormerod, F. D'A. M. Williams, H. B. Wilson.

**Royal College of Surgeons.**—First Professional Examination in Anatomy and Physiology for the diploma of Fellow, Nov. 11.—A. E. H. Pinch, M.R.C.S., L.R.C.P.; H. T. S. Aveline, M.R.C.S., L.R.C.P.

**Society of Apothecaries.**—September. *Forensic Medicine*—A. H. Grace.

### HOSPITAL FOR SICK CHILDREN AND WOMEN.

W. Barrett Roué, M.D., M.S. Durh., has resigned his position on the active staff, and been appointed Consulting Physician.

E. Leonard Lees, M.D., C.M. Edin., has been appointed Physician.

W. M. Willis, M.R.C.S. Eng., L.R.C.P. Lond., has been appointed House Physician.

### EYE HOSPITAL.

C. H. Walker, B.A., M.B. Cantab., F.R.C.S. Eng., has been appointed Assistant Surgeon.

### LIST OF SUBSCRIBERS

TO

### The Bristol Medico-Chirurgical Journal.

*DECEMBER*, 1895.

A. W. Peake

W. J. Penny

C. F. Pickering

A. E. H. Pinch

W. E. Pountney

J. J. Powell

A. Prichard

A. W. Prichard

J. E. Prichard

A. Pring

A. B. Prowse

W. Richardson

### GLOUCESTERSHIRE *(Continued).*

BRISTOL *(continued).*

B. M. H. Rogers

J. Rendall

SOUTHAMPTON.

E. Blacker

W. R. Y. Ives

SOUTHSEA.

J. W. Cousins

VENTNOR.

E. R. Woodford

### HERTFORDSHIRE.

ST. ALBAN'S.

A. H. Boys

### HUNTINGDONSHIRE.

ST. NEOT'S.

J. C. Grey

J. H. Hemming

### LANCASHIRE.

LIVERPOOL.

G. A. Hawkins-Ambler
Medical Institution
C. Williams

POULTON-LE-FYLDE.

P. H. Day

PRESTON.

J. E. Garner

### MIDDLESEX.

C. Harrison

G. G. D. Willett

### SOMERSETSHIRE *(Continued).*

LANGPORT.

J. Morgan

MIDSOMER NORTON.

A. Waugh

A. H. Whicher

MILVERTON.

C. Randolph

NORTH PETHERTON.

C. F. Hawkins

PILL.

T. W. S. Morgan

PORTISHEAD.

W. Monckton

C. A. Wigan

SHEPTON MALLET.

J. T. Hyatt

SOUTH PETHERTON.

S. W. MacIlwaine

TAUNTON.

Taunton Hospital

J. A. Macdonald

H. T. Rutherford

TIMSBURY.

F. Woods

WEDMORE.

J. Ford

R. P. Tyley

WELLINGTON.

J. Meredith

WELLS.

F. J. B. Bateman

W. Fairbanks

A. L. Wade

WESTON-SUPER-MARE.

G. E. Alford

C. P. Crouch

G. B. Fraser

E. F. Martin

W. H. G. Phelps

G. F. Rossiter

R. Roxburgh

J. Wallace

R. Wilde

WORLE.

F. St. J. Kemm

WRINGTON.

A. H. Benson

H. W. Collins

YEOVIL.

P. S. H. Colmer

### STAFFORDSHIRE.

STAFFORD.
J. Scott

### SURREY.

EPSOM.
R. Davis

ESHER.
J. B. Fry

VIRGINIA WATER.
S. R. Philipps

REDHILL.

C. M. Jessop

### SUSSEX.

ST. LEONARD'S-ON-SEA.
W. H. Spencer

### WARWICKSHIRE.

LEAMINGTON.
A. W. W. Hoffman

### WILTSHIRE.

BRADFORD-ON-AVON.

W. J. A. Adye.

J. Beddoe

CALNE.

W. S. Batten

W. A. Hayes

DEVIZES.
T. B. Anstie
C. Eddowes
A. M. Gray
H. J. Mackay

FOVANT.
C. Clay

MALMESBURY.
R. Kinneir

MERE.

J. McElfatrick

PEWSEY.

H. Colman

SALISBURY.

L. S. Luckham

SWINDON.

W. Howse

J. C. Maclean

WARMINSTER.

R. L. Willcox

### YORKSHIRE.

HARROGATE.

A. O. Jones

SHEFFIELD.

S. Snell

### Scotland.

### EDINBURGHSHIRE.

EDINBURGH.
J. W. Ballantyne   Y. J. Pentland

### Ireland.

### ANTRIM.

BELFAST.

J. W. Browne   A. Jamison   J. A. Lindsay

### DUBLIN.

DUBLIN.

G. Carolin

### Wales.

### BRECONSHIRE.

BRECON.
A. Whyte

SENNY BRIDGE.

W. R. Jones

TALGARTH.

W. Howells

### CARMARTHENSHIRE.

CARMARTHEN.

R. G. Price

FERRYSIDE.

P. Williams

LLANELLY.

S. J. Roderick

D. J. Williams

### CARNARVONSHIRE.

ST. CLEARS.
R. Thomas

### GLAMORGANSHIRE.

ABERAMAN.

J. B. James

ABERDARE.

E. Jones

CAERPHILLY.

J. Lewellyn

CARDIFF.

E. T. Collins

W. T. Edwards

C. A. Griffiths

P. R. Griffiths

J. Milward

D. R. Paterson

J. A. Phillips

W. Price

A. Rees

A. Sheen

Medical Society

J. L. Thomas

H. R. Vachell

J. Williams

COWBRIDGE.

T. R. Hamlen-Williams

MAESTEG.

W. H. Thomas

MERTHYR TYDVIL.

W. W. Jones

C. E. G. Simons

J. L. W. Ward

PENARTH.
R. F. Nell

PORTH.

H. N. Davies

SWANSEA.

E. Davies   R. C. Elsworth

TREHARRIS.

W. W. Leigh

### PEMBROKESHIRE.

FISHGUARD.

H. L. Swete

HAVERFORDWEST.

E. P. Phillips

PENALLY.

B. C. Kendall

### RADNORSHIRE.

LLANDRINDOD WELLS.
A. G. Greenway

### Belgium.

COURTRAI.

Dr. Lauwers

### Italy.

GENOA.

S. Bramonti

### Switzerland.

DAVOS-PLATZ.

W. R. Huggard

### Victoria.

BRADFORD.

G. H. Skinner

### Cape Colony.

FORT BEAUFORT.

J. Shaw

### Egypt.

ALEXANDRIA.

J. Mackie

### United States.

CINCINNATI.

Cincinnati Hospital

COLORADO.

S. E. Solly

### New Zealand.

LOWER HUTT.

J. R. Purdy

### INDEX.

Abdominal lesions, Treatment of, 206.
Abdominal tumours and abdominal dropsy in women—Dr. J. Oliver (*Review*), 220.
Accouchement, Considérations sur les ruptures de l'utérus et du vagin pendant l'—Dr. G. I. Andronesco (*Review*), 302.
Ætiology of disease, The—Dr. P. H. Pye-Smith (*Review*), 301.
Africa, Tropical diseases in—Dr. R. W. Felkin (*Review*), 223.
Album, The (*Review*), 64.
Alcoholic disease of the heart—Dr. T. Fisher, 81.
Allingham bobbin in intestinal anastomosis, 206.
Alpine climates for consumption—Dr. H. J. Hardwicke (*Review*), 61.
American Surgical Association, Transactions of the (*Review*), 230.
Anæmia, Sclerosis in, 120.
Anæsthesia, Inventor of, 148.
Anatomy and art—Dr. R. Fletcher (*Review*), 224.
Anatomy of the nasal cavity, The —Dr. A. Ónodi (*Review*), 214.
Anderson, Dr. J.—Medical nursing (*Review*), 50.
Anderson, Dr. T. M'C.—A treatise on diseases of the skin (*Review*), 46.
Andronesco, Dr. G. I.—Considérations sur les ruptures de l'utérus et du vagin pendant l'accouchement et la valeur de la laparotomie dans leur traitement (*Review*), 302.
Antiseptic midwifery, 139.
Antitoxin treatment of diphtheria, 24, 197.
Anus and fæcal fistula, Extra-peritoneal closure of artificial—J. G. Smith, 1.
Anus, The rectum and—C. B. Ball (*Review*), 55.
Arsenic in skin diseases, 212.
Art, Anatomy and—Dr. R. Fletcher (*Review*), 224.
Aseptic treatment of wounds, The —Dr. C. Schimmelbusch (*Review*), 219.
Asthma and chronic bronchitis—Dr. J. C. Thorowgood (*Review*), 64.
Atheroma, Pathology of, 28.
Auto-intoxication in disease—C. Bouchard (*Review*), 48.

Bacteriological examination of diphtheria cultures, 320.
Bacteriologist, Laboratory guide for the — L. Frothingham (*Review*), 225.
Bacteriology, 117, 121, 122, 138, 196.
Bacterioscopic diagnosis of diphtheria, The—F. W. Stoddart, 18.
Baldy, Dr. J. M. [Ed.]—An American text-book of gynecology (*Review*), 38.
Ball, C. B.—The rectum and anus (*Review*), 55.
Ball, Dr. J. B.—Diseases of the nose and pharynx (*Review*), 42.
Barclay, W. M.—Report on surgery, 29.
Baron, Dr. B. J.—Report on laryngology, 286.
Bassin, De l'agrandissement momentané du—A. Pinard (*Review*), 50.
Beach, Dr. F.—The treatment and education of mentally feeble children (*Review*), 303.
Bentley, Dr. A. J. M.—Bèri-bèri (*Review*), 41.
Bentley and Rev. C. G. Griffinhoofe, Dr. A. J. M.—Wintering in Egypt (*Review*), 224.
Bèri-bèri—Dr. A. J. M. Bentley (*Review*), 41.
Bigelow, Dr. H. R. [Ed.] — An international system of electrotherapeutics (*Review*), 39.
Black, Dr. D. C.—The urine in health and disease, and urinary analysis (*Review*), 143.
Bladder tumours, Cystotomy for—J. P. Bush, 8.

Blake, Dr. E. T. — Myxœdema, cretinism, and the goitres (*Review*), 62.
Blood-poisoning in brothers — Dr. F. H. Edgeworth, 107.
Booksellers, Directory of second-hand (*Review*), 64.
Bouchard, C.—Lectures on auto-intoxication in disease (*Review*), 48.
Braidwood, Dr. P. M.—The mother's help and guide to the domestic management of her children (*Review*), 226.
Brain, The insanity of over-exertion of the — Dr. J. B. Tuke (*Review*), 141.
Breast, Cancer of the, 29.
Breast, Diseases of the — W. R. Williams (*Review*), 52.
Bristol health reports, 237, 320.
Bristol Medico-Chirurgical Society, 68, 151, 312.
Brodie, C. G. — Dissections illustrated (*Review*), 63, 301.
Bronchitis, Asthma and chronic—Dr. J. C. Thorowgood (*Review*), 64.
Browne, R.—Neurasthenia and its treatment by hypodermic transfusions (*Review*), 56.
Bubis, Dr. G.—Sperminum-Poehl in chemischer, physiologischer, und therapeutischer beziehung (*Review*), 61.
Buccal glandular tumours—Dr. J. Swain, 94.
Burdett, H. C.—Helps in sickness and to health (*Review*), 146.
Bush, J. P.—Cystotomy for bladder tumours of doubtful malignancy, 8.
Bush, Dr. J. E. Shaw and J. P.—Sarcomatous tumour of the shoulder-centre in the cortex of the right hemisphere, 99.

Cancer and of simple dilatation of the stomach, On the diagnosis of —Dr. J. M. Clarke, 181.
Cancer of the breast, 29.
Castration in prostatic hypertrophy, 125.
Cerna, Dr. D.—Notes on the newer remedies (*Review*), 226.
Cervix uteri, Lacerations of the, 134.
Chemistry, Cod-liver oil and—F. P. Möller (*Review*), 215.
Cheyne, W. W.—The treatment of wounds, ulcers, and abscesses (*Review*), 47.
Chicago, Annual report of the department of health of the city of (*Review*), 306.
Children, Mentally feeble—Dr. F. Beach (*Review*), 303.
Children, Prescribing and treatment for infants and — P. E. Muskett (*Review*), 61.
Children, The mother's help and guide to the domestic management of her —Dr. P. M. Braidwood (*Review*), 226.
Children, The surgical diseases of—D'A. Power (*Review*), 296.
Chisholm, Dr. J. E. Pollock and J.—Medical handbook of life assurance (*Review*), 304.
Cholera expenses, State aid in relation to Port—E. Hart (*Review*), 60.
Cholera, The nurseries of—E. Hart (*Review*), 62.
Clarke, Dr. J. M.—On the diagnosis of cancer and of simple dilatation of the stomach, 181; Report on medicine, 273.
Clinical manual, A—Dr. A. MacFarlane (*Review*), 63.
Clinical sketches (*Review*), 60.
Clinical Society of London, Transactions of the (*Review*), 147.
Cod-liver oil and chemistry — F. P. Möller (*Review*), 215.
Consumption, Alpine climates for—Dr. H. J. Hardwicke (*Review*), 61.
Consumption, Prevention of — Dr. W. Murrell (*Review*), 304.
Cooper, A.—Syphilis (*Review*), 294.
Cretinism, and the goitres, Myxœdema—Dr. E. T. Blake (*Review*), 62.
Crookshank, Dr. E. M.—The prevention of small-pox (*Review*), 227.
Cross, F. R.—Report on ophthalmology, 34.
Crouch, Dr. C. P.—Some clinical notes on membranous enteritis, 14.
Cystotomy for bladder tumours—J. P. Bush, 8.

DaCosta, Dr. J. C.—A manual of modern surgery (*Review*), 142.
Davey, Obituary notice of Dr. J. G., 76.
Davies, A. M — A handbook of hygiene (*Review*), 298.
Deaf-mutism, 289; Dr. H. Mygind (*Review*), 45.
Dermatology, Report on — Dr. H. Waldo, 207.
Devon and Exeter Medico-Chirurgical Society, 72, 152.
Diabetes—Dr. A. Vintras (*Review*), 302.

Diet lists—Dr. J. B. Thomas (*Review*), 303.
Diphtheria and its treatment, 68; Dr. B. R. Martin (*Review*), 225.
Diphtheria, Antitoxin treatment of, 24, 197.
Diphtheria cultures, Bacteriological examination of, 320.
Diphtheria, The bacterioscopic diagnosis of—F. W. Stoddart, 18.
Directory of second-hand booksellers (*Review*), 64.
Dissections illustrated—C. G. Brodie (*Review*), 63, 301.
Droitwich brine baths, The—W. H. Tomlins (*Review*), 227.
Dührssen, Dr. A.—A manual of gynæcological practice (*Review*), 295.
Dynamics of life, The—Dr. W. R. Gowers (*Review*), 146.
Dyspepsia in women, The common forms of—Dr. R. Saundby (*Review*), 55.
Dyspepsia of phthisis, The—Dr. W. S. Fenwick (*Review*), 51.

Ear, Diseases of the—Dr. E. B. Gleason (*Review*), 62; T. M. Hovell (*Review*), 37; Dr. A. M. Sheild (*Review*), 220.
Edgeworth, Dr. F. H.—Four cases of blood-poisoning in brothers, 107.
Edinburgh hospital reports (*Review*), 60.
Egypt as a health-resort—Dr. P. W. Williams, 263.
Egypt, Wintering in—Dr. A. J. M. Bentley and Rev. C. G. Griffinhoofe (*Review*), 224.
Electro-therapeutics, An international system of—Dr. H. R. Bigelow [Ed.] (*Review*), 39.
Embryology, Syllabus of lectures on human—Dr. W. P. Manton (*Review*), 145.
Enteric fever from eating oysters, 25.
Enteritis, Some clinical notes on membranous—Dr. C. P. Crouch, 14.
Epidemiological Society of London, Transactions of the (*Review*), 305.
Epilepsy, 273.
Erysipelas toxin treatment of malignant growths, 312.
Ether, The administration of—J. Freeman, 191.
Ether, The administration of nitrous oxide before—Dr. B. M. H. Rogers, 10.
Eye, nose, and throat, Diseases of—Drs. E. Jackson and E. B. Gleason (*Review*), 58.

Fawcett, Prof. E.—On the localisation of the foramina at the base of the skull, 173.
Feachem, M.—Food for the sick and convalescent (*Review*), 227.
Felkin, Dr. R. W.—On the geographical distribution of tropical diseases in Africa (*Review*), 223.
Femur, Fractures of the upper third of the, 202.
Fenwick, E. H.—Urinary surgery (*Review*), 225.
Fenwick, Dr. W. S.—The dyspepsia of phthisis (*Review*), 51.
First aid to the injured and management of the sick—Dr. E. J. Lawless (*Review*), 63.
Fisher, Dr. T.—Alcoholic disease of the heart, 81; Report on medicine, 24; Two cases of fibroid degeneration of the myocardium, 258.
Fletcher, Dr. R.—Anatomy and art (*Review*,) 224.
Food for the sick and convalescent—M. Feachem (*Review*), 227.
Foot, The deformities of the human—W. J. Walsham and W. K. Hughes (*Review*), 294.
Foramina at the base of the skull, On the localisation of the—Prof. E. Fawcett, 173.
Foxwell, Dr. A.—Essays in heart and lung disease (*Review*), 292.
Fractures of the upper third of the femur, 202.
Freeman, J.—The administration of ether, 191.
Frothingham, L.—Laboratory guide for the bacteriologist (*Review*), 225.

Gleason, Dr. E. B.—Essentials of diseases of the ear (*Review*), 62.
Gleason, Drs. E. Jackson and E. B.—Essentials of diseases of eye, nose, and throat (*Review*), 58.
Goitre, Surgical treatment of exophthalmic, 280.
Goitres, Myxœdema, cretinism, and the—Dr. E. T. Blake (*Review*), 62.
Gout—Dr. R. Roose (*Review*), 56.
Gowers, Dr. W. R.—The dynamics of life (*Review*), 146.
Grandin and G. W. Jarman, Drs. E. H.—Obstetric surgery (*Review*), 298.
Griffinhoofe, Dr. A. J. M. Bentley and Rev. C. G.—Wintering in Egypt (*Review*), 224.

Griffiths, L. M.—Report on obstetrics, 134.
Gynæcological practice, A manual of—Dr. A. Dührssen (*Review*), 295.
Gynecology, An American text-book of—Dr. J. M. Baldy [Ed.] (*Review*), 38.
Gynecology, Syllabus of—Dr. J. W. Long (*Review*), 226.

Hall, Dr. F. De H.—Diseases of the nose and throat (*Review*), 44.
Hardwicke, Dr. H. J. — Alpine climates for consumption (*Review*), 61.
Harrogate mineral waters, The—Dr. A. Roberts (*Review*), 56.
Harsant, W. H.—Report on otology, 289; Report on surgery, 280.
Hart, E.—Compulsory notification in England and Wales in 1892. State aid in relation to Port cholera expenses (*Review*), 60; Compulsory vaccination, and small-pox in relation to vaccination (*Review*), 60; The nurseries of cholera (*Review*), 62.
Harvey, Dr. W.—An anatomical dissertation upon the movement of the heart and blood in animals, being a statement of the discovery of the circulation of the blood (*Review*), 301.
Health, Helps in sickness and to—H. C. Burdett (*Review*), 146.
Heart, Alcoholic disease of the—Dr. T. Fisher, 81.
Heart and lung disease, Essays in—Dr. A. Foxwell (*Review*), 292.
Heart disease, Clinical diagrams for —Dr. G. Herschell (*Review*), 59.
Heart, The Schott methods of the treatment of chronic diseases of the—Dr. W. B. Thorne (*Review*), 293.
Hellier, Dr. J. B. — Infancy and infant-rearing (*Review*), 296.
Hermes medical supplement (*Review*), 309.
Herpes, Morphœa and, 211.
Herschell, Dr. G.—Clinical diagrams, with directions for recording cases of heart disease (*Review*), 59; Indigestion (*Review*), 293.
History from the earliest times, Medical—Dr. E. T. Withington (*Review*), 141.
Hospital annual, Burdett's (*Review*), 308.
Hovell, T. M.—Diseases of the ear (*Review*), 37.
Hughes, W. J. Walsham and W. K. —The deformities of the human foot (*Review*), 294.
Hutton, A. W. — The vaccination question (*Review*), 221.
Hydatid disease—Dr. J. D. Thomas (*Review*), 43.
Hygiene, A handbook of — A. M. Davies (*Review*), 298.
Hygiene, Text-book of—Dr. G. H. Rohé (*Review*), 145.

Index-catalogue of Library of the Surgeon-General's Office, United States Army (*Review*), 230.
Indigestion—Dr. G. Herschell (*Review*), 293.
Inebriety or narcomania—Dr. N. Kerr (*Review*), 59.
Infancy and infant-rearing—Dr. J. B. Hellier (*Review*), 296.
Inhalation — Dr. W. H. Spencer (*Review*), 303.
Insanity, Nervous diseases and—Dr. J. C. Shaw (*Review*), 56.
Insanity of over-exertion of the brain, The—Dr. J. B. Tuke (*Review*), 141.
Intestinal anastomosis, Murphy button in, 204; Allingham bobbin in, 206.
Ireland, Transactions of the Royal Academy of Medicine in (*Review*), 306.

Jackson and E. B. Gleason, Drs. E. —Essentials of diseases of eye, nose, and throat (*Review*), 58.
Jarman, Drs. E. H. Grandin and G. W. — Obstetric surgery (*Review*), 298.
Jeaffreson, Dr. C. S.—Notes on nursing in eye diseases (*Review*), 58.
Jenks memorial prize, 237.
Johns Hopkins hospital reports (*Review*), 228, 304.

Kerr, J. G.—Vocabulary of diseases (*Review*), 308.
Kerr, Dr. N.—Inebriety or narcomania (*Review*), 59.

Lacerations of the cervix uteri, 134.
La Garriga, Característica terapéutica de las aguas termales de—Dr. M. Manzaneque (*Review*), 59.
Laryngeal tuberculosis, Surgical treatment of, 286.
Laryngology, Report on—Dr. B. J. Baron, 286.

Larynx and trachea, External examination of, 287.
Lawless, Dr. E. J.—First aid to the injured and management of the sick (*Review*), 63.
Library of the Bristol Medico-Chirurgical Society, 73, 153, 234, 316.
Library of the Surgeon-General's Office, United States Army, Index-catalogue of (*Review*), 230.
Life assurance, Medical handbook of—Dr. J. E. Pollock and J. Chisholm (*Review*), 304.
Life, The dynamics of—Dr. W. R. Gowers (*Review*), 146.
Local medical notes, 158, 237, 320.
Long, Dr. J. W.—Syllabus of gynecology, based on the American text-book of gynecology (*Review*), 226.
Lunacy law for medical men—Dr. C. Mercier (*Review*), 45.
Lung disease, Essays in heart and—Dr. A. Foxwell (*Review*), 292.
Lupus of the nose and palate, 288.
Lupus vulgaris, 211.

MacFarlane, Dr. A. — A clinical manual (*Review*), 63.
Mackay, Dr. G.—On blinding of the retina by direct sunlight (*Review*), 58.
Maddox, Dr. E. E.—The clinical use of prisms and the decentering of lenses (*Review*), 57.
Male nurses, 234.
Manton, Dr. W. P.—Syllabus of lectures on human embryology (*Review*), 145.
Manzaneque, Dr. M.—Característica terapéutica y especialización clínica de las aguas termales de La Garriga (*Review*), 59.
Martin, Dr. B. R.—Diphtheria and its treatment (*Review*), 225.
Martindale, W.—Analyses of twelve thousand prescriptions (*Review*), 55.
Martindale and Dr. W. W. Westcott, W.—The extra pharmacopœia (*Review*), 303.
Medical annual (*Review*), 230.
Medical history from the earliest times — Dr. E. T. Withington (*Review*), 141.
Medical Society of London, Transactions of the (*Review*), 229.
Medicine, Bacteriology in, 117, 121, 122, 196.
Medicine, Reports on—Dr. J. M. Clarke, 273; Dr. T. Fisher, 24; Dr. G. Parker, 117; Dr. R. S. Smith, 196.
Medicine, The theory and practice of—Dr. F. T. Roberts (*Review*), 52.
Medico-Chirurgical Transactions (*Review*), 229.
Membranous enteritis, Some clinical notes on—Dr. C. P. Crouch, 14.
Mercier, Dr. C.—Lunacy law for medical men (*Review*), 45.
Middlesex hospital reports (*Review*), 228.
Möller, F. P.—Cod-liver oil and chemistry (*Review*), 215.
Morphœa and herpes, 211.
Morton, C. A.—Report on surgery, 125.
Moullin, Dr. C. W. M.—Enlargement of the prostate (*Review*), 54.
Murphy button in intestinal anastomosis, 204.
Murrell, Dr. W.—Clinical lectures on the prevention of consumption (*Review*), 304.
Muskett, P. E. — Prescribing and treatment for infants and children (*Review*), 61.
Mygind, Dr. H. — Deaf-mutism (*Review*), 45.
Myocardium, Fibroid degeneration of the—Dr. T. Fisher, 258.
Myxœdema, cretinism, and the goitres—Dr. E. T. Blake (*Review*), 62.

Narcomania, Inebriety or—Dr. N. Kerr (*Review*), 59.
Nasal cavity, The anatomy of the—Dr. A. Ónodi (*Review*), 214.
Nasal polypi, 288.
Nerve-diseases, Etiology of, 118.
Nervous diseases and insanity—Dr. J. C. Shaw (*Review*), 56.
Neurasthenia and its treatment by hypodermic transfusions — R. Browne (*Review*), 56.
Nitrous oxide before ether, The administration of—Dr. B. M. H. Rogers, 10
Nose and pharynx, Diseases of the—Dr. J. B. Ball (*Review*), 42.
Nose, and throat, Diseases of eye—Drs. E. Jackson and E. B. Gleason (*Review*), 58.
Nose and throat, Diseases of the—Dr. F. De H. Hall (*Review*), 44.
Notification in England and Wales in 1892, Compulsory—E. Hart (*Review*), 60.
Nurses, Male, 234.
Nursing in eye diseases, Notes on—Dr. C. S. Jeaffreson (*Review*), 58.
Nursing, Medical—Dr. J. Anderson (*Review*), 50.

Nursing, Ophthalmic — Dr. S. Stephenson (*Review*), 57.

Obstetric surgery — Drs. E. H. Grandin and G. W. Jarman (*Review*), 298.
Obstetrics, Bacteriology in, 138.
Obstetrics, Report on — L. M. Griffiths, 134.
Oliver, Dr. G.— Pulse - gauging (*Review*), 144.
Oliver, Dr. J.—Abdominal tumours and abdominal dropsy in women (*Review*), 220.
Ónodi, Dr. A.—The anatomy of the nasal cavity and its accessory sinuses (*Review*), 214.
Ophthalmia neonatorum, 34.
Ophthalmological Society of the United Kingdom, Transactions of the (*Review*), 63.
Ophthalmology, Report on—F. R. Cross, 34.
Osteo-arthritis, The paralysis of—Dr. H. Waldo, 90.
Os uteri, Treatment of rigid, 140.
Otology, Report on—W.H. Harsant, 289.
Oxygen in skin diseases, 213.
Oysters, Infection with enteric fever by eating, 25.

Paracentesis of the pericardium, 26.
Paralysis of osteo-arthritis—Dr. H. Waldo, 90.
Parker, Dr. G.—Report on medicine, 117.
Pathology, Elements of surgical—Dr. A. J. Pepper (*Review*), 64.
Pepper, Dr. A. J.—Elements of surgical pathology (*Review*), 64.
Pericardium, Paracentesis of the, 26.
Pharmacopœia of the hospital for diseases of the throat (*Review*), 63.
Pharmacopœia, The extra—W. Martindale and Dr. W. W. Westcott (*Review*), 303.
Pharmacy, Practice of—L. E. Sayre (*Review*), 226.
Pharynx, Diseases of the nose and —Dr. J. B. Ball (*Review*), 42.
Phthisis, The dyspepsia of—Dr. W. S. Fenwick (*Review*), 51.
Physiology, A manual of human—Dr. J. H. Raymond (*Review*), 48.
Pinard, A — De l'agrandissement momentané du bassin (*Review*), 50.
Pneumococci in the urine—G. M. Smith, 115.
Pneumonia, 199.
Pollock and J. Chisholm, Dr. J. E. —Medical handbook of life assurance (*Review*), 304.
Power, D'A.—The surgical diseases of children, and their treatment by modern methods (*Review*), 296.
Practitioner, The (*Review*), 231.
Pregnant women, Care of, 137.
Prescriptions, Analyses of twelve thousand—W. Martindale (*Review*), 55.
Prichard, A. W.—School surgery, 241.
Prisms, The clinical use of—Dr. E. E. Maddox (*Review*), 57.
Prostate, Enlargement of the.—Dr. C. W. M. Moullin (*Review*), 54.
Prostatic hypertrophy, Castration in, 125.
Pruritus, 213.
Psoriasis, Internal therapeutics of, 210.
Pulse-gauging—Dr. G. Oliver (*Review*), 144.
Purdy, Dr. C. W.—Practical uranalysis and urinary diagnosis (*Review*), 145.
Pye-Smith, Dr. P. H.—The ætiology of disease (*Review*), 301.

Raymond, Dr. J. H.—A manual of human physiology (*Review*), 48.
Rectum and anus, The—C. B. Ball (*Review*), 55.
Remedies, The newer — Dr. D. Cerna (*Review*), 226.
Respiratory tract, Diseases of the upper—Dr. P. W. Williams (*Review*), 49.
Retina by direct sunlight, On blinding of the—Dr. G. Mackay (*Review*), 58.
Rheumatism, Acute, 121, 201.
Roberts, Dr. A.—The Harrogate mineral waters (*Review*), 56.
Roberts, Dr. F. T.—The theory and practice of medicine (*Review*), 52.
Rogers, Dr. B. M. H.—The administration of nitrous oxide as a preliminary to ether anæsthesia, 10.
Rohé, Dr. G. H.—Text-book of hygiene (*Review*), 145.
Roose, Dr. R.—Gout (*Review*), 56.

Saint Bartholomew's hospital reports (*Review*), 305.
Saint Thomas's hospital reports (*Review*), 147.
Sarcomatous tumour of the shoulder-centre—Dr. J. E. Shaw and J. P. Bush, 99.
Saundby, Dr. R.—The common forms of dyspepsia in women (*Review*), 55.

Sayre, L. E.—Essentials of practice of pharmacy (*Review*), 226.
Scabies, 212.
Schimmelbusch, Dr. C.—The aseptic treatment of wounds (*Review*), 219.
School surgery—A. W. Prichard, 241.
Schott methods of the treatment of chronic diseases of the heart, The —Dr. W. B. Thorne (*Review*), 293.
Sclerosis in anæmia, 120.
Scraps, 79, 159, 239, 323.
Shaw, Dr. J. C.—Essentials of nervous diseases and insanity (*Review*), 56.
Shaw and J. P. Bush, Dr. J. E.—Sarcomatous tumour of the shoulder-centre in the cortex of the right hemisphere, 99.
Sheild, Dr. A. M.—Diseases of the ear (*Review*), 220.
Shoulder-centre, Sarcomatous tumour of the—Dr. J. E. Shaw and J. P. Bush, 99.
Sickness and to health, Helps in—H. C. Burdett (*Review*), 146.
Sick, Preparations for the, 65, 149, 231, 309.
Skin, Diseases of the—Dr. T. M'C. Anderson (*Review*), 46.
Skull, On the localisation of the foramina at the base of the—Prof. E. Fawcett, 173.
Small-pox, The prevention of—Dr. E. M. Crookshank (*Review*), 227.
Smith, G. M.—Pneumococci in the urine, 115.
Smith, J. G. — Extra - peritoneal closure of artificial anus and fæcal fistula, 1.
Smith, Dr. R. S.—Report on medicine, 196.
Society, Bristol Medico-Chirurgical, 68, 151, 312.
Society, Devon and Exeter Medico-Chirurgical, 72, 152.
Speech, The disorders of—Dr. J. Wyllie (*Review*), 216.
Spencer, Dr. W. H.—A new method of inhalation for the treatment of diseases of the lungs (*Review*), 303.
Sperminum-Poehl in chemischer, physiologischer, und therapeutischer beziehung—Dr. G. Bubis (*Review*), 61.
Spine, Diseases and deformities of the—R. L. Swan (*Review*), 143.
Stephenson, Dr. S. — Ophthalmic nursing (*Review*), 57.
Sterilisation of dressings in eye diseases, 37.
Stevens, Dr. A. A.—A manual of therapeutics (*Review*), 44.
Stoddart, F. W.—The bacterioscopic diagnosis of diphtheria, 18.
Stomach, On the diagnosis of cancer and of simple dilatation of the—Dr. J. M. Clarke, 181.
Sunlight, On blinding of the retina by direct—Dr. G. Mackay (*Review*), 58.
Surgery, A manual of modern—Dr. J. C. DaCosta (*Review*), 142.
Surgery in the last twenty-five years, The progress of—H. P. Symonds, 161.
Surgery, Reports on—W. M. Barclay, 29; W. H. Harsant, 280; C. A. Morton, 125; Dr. J. Swain, 202.
Surgical pathology, Elements of—Dr. A. J. Pepper (*Review*), 64.
Swain, Dr. J.—Buccal glandular tumours, 94; Report on surgery, 202.
Swan, R. L.—Manual of diseases and deformities of the spine (*Review*), 143.
Swayne, Presentation to Dr. J. G., 78.
Symonds, H. P.—The progress of surgery in the last twenty-five years, 161.
Syphilis—A. Cooper (*Review*), 294.

Therapeutic notes, Pocket (*Review*), 65.
Therapeutics, A manual of—Dr. A. A. Stevens (*Review*), 44.
Thomas, Dr. J. B.—A book of detachable diet lists (*Review*), 303.
Thomas, Dr. J. D.—Hydatid disease (*Review*), 43.
Thorne, Dr. W. B.—The Schott methods of the treatment of chronic diseases of the heart (*Review*), 293.
Thorowgood, Dr. J. C.—Asthma and chronic bronchitis (*Review*), 64.
Throat, Diseases of eye, nose, and —Drs. E. Jackson and E. B. Gleason (*Review*), 58.
Throat, Diseases of the nose and —Dr. F. De H. Hall (*Review*), 44.
Throat, Pharmacopœia of the hospital for diseases of the (*Review*), 63.
Thyroid gland, Surgical treatment of tumours of the, 203.
Tomlins, W. H.—The Droitwich brine baths as therapeutic agents in various diseases (*Review*), 227.
Tropical diseases in Africa—Dr. R. W. Felkin (*Review*). 223.

Tuke, Dr. J. B.—The insanity of over-exertion of the brain (*Review*), 141.

Uranalysis and urinary diagnosis, Practical — Dr. C. W. Purdy (*Review*), 145.
Uric acid diathesis, 124.
Urinary surgery — E. H. Fenwick (*Review*), 225.
Urine in health and disease, The — Dr. D. C. Black (*Review*), 143.
Urine, Pneumococci in the—G. M. Smith, 115.

Vaccination, Compulsory vaccination, and small-pox in relation to —E. Hart (*Review*), 60.
Vaccination question, The—A. W. Hutton (*Review*), 221.
Vintras, Dr. A.—Diabetes, and its treatment (*Review*), 302.
Visiting list (*Review*), 231.
Vocabulary of diseases—J. G. Kerr (*Review*), 308.
Vocal defects in teachers, 289.

Waldo, Dr. H.—Report on dermatology, 207; The paralysis of osteo-arthritis, 90.
Walsham and W. K. Hughes, W. J.—The deformities of the human foot (*Review*), 294.
Westcott, W. Martindale and Dr. W. W.—The extra pharmacopœia (*Review*), 303.
Westminster hospital reports (*Review*), 229.
Williams, Dr. P. W. — Diseases of the upper respiratory tract (*Review*), 49; Notes on Egypt as a health-resort, 263.
Williams, W. R.— Diseases of the breast (*Review*), 52.
Withington, Dr. E. T.— Medical history from the earliest times (*Review*), 141.
Women, Abdominal tumours and abdominal dropsy in — Dr. J. Oliver (*Review*), 220.
Women, The common forms of dyspepsia in — Dr. R. Saundby (*Review*), 55.
Workhouses, The sick poor in (*Review*), 227.
Wounds, The aseptic treatment of— Dr. C. Schimmelbusch (*Review*), 219.
Wounds, ulcers, and abscesses, The treatment of — W. W. Cheyne (*Review*), 47.
Wyllie, Dr. J.—The disorders of speech (*Review*), 216.

Year-book of scientific societies (*Review*), 308.
Year-book of treatment (*Review*), 230.

J. W. Arrowsmith, Printer, Quay Street, Bristol.

